# Predicting the degree of trait emotional empathy from cortical features using surface-based morphometry

**DOI:** 10.1038/s41598-026-44137-9

**Published:** 2026-03-25

**Authors:** Lukas Novak, Klara Malinakova, Jitse P. van Dijk, Peter Tavel, William Penny, Petr Hluštík

**Affiliations:** 1 OUSHI - Olomouc University Social Health Institute, Palacký University Olomouc, Univerzitni 244/22, Olomouc, 771 11 Czech Republic; 2 Department of Community and Occupational Medicine, University Medical Center Groningen, University of Groningen, Groningen, The Netherlands; 3 Graduate School Kosice Institute for Society and Health, P.J. Safarik University in Kosice, Kosice, Slovak Republic; 4 School of Psychology, University of East Anglia, Norwich, UK; 5 Department of Neurology, Faculty of Medicine and Dentistry, Palacký University, Olomouc, Czech Republic

**Keywords:** Empathy, MRI, Neural basis, Machine learning, Surface-based morphometry, Sulcus depth, Cortical thickness, Gyrification, SPLSR, Neuroscience, Psychology, Psychology

## Abstract

The link between neuroanatomy and empathy was a primary aim of many studies in the past. However, these studies are often limited by partial volume effects inherent to Voxel-Based Morphometry (VBM), which hinder the accurate assessment of cortical features. To overcome these limitations, we used Surface-Based Morphometry (SBM) to investigate the relationship between cortical features such as sulcal depth and trait emotional empathy for the first time. We also tested several hypotheses based on previous research. Our study sample consisted of 62 adults. Trait empathy was measured by the Toronto Empathy Questionnaire. We used methods from machine learning (e.g., Leave-one-out cross-validation – LOO-CV – in conjunction with Sparse Partially Least Squares regression - SPLSR) to evaluate whether cortical features (i.e., gyrification, sulcal depth, and cortical thickness) could accurately predict the degree of trait emotional empathy. We tested the hypotheses of the present study using Linear Mixed Effects models. The Regions of Interest (ROI) tested within these hypotheses included bilateral insula and dorsal Anterior Cingulate Cortex (dACC). A significant negative association was found between the cortical thickness of the left insula and trait emotional empathy score (B = -4.72; 95% CI [-8.52, -0.92]; *p* = 0.019). In addition, there was a negative association between cortical thickness in the left dACC and trait emotional empathy (B = -5.47; 95% CI [-9.48, -1.46]; *p* = 0.008). The SPLSR models showed moderate training fit (${R^2}$ ≈ 60% in gyrification) but failed to generalize to unseen data, with negative cross-validated ${Q^2}$ values across all 3 cortical features. While ROI analyses revealed significant negative associations between cortical thickness and trait emotional empathy, machine learning models failed to achieve reliable out-of-sample prediction, likely due to the high-dimensional feature space relative to sample size. The predictive findings should therefore be considered exploratory. Future studies should use larger samples and explore how differences in white matter structure can explain individual differences in empathy.

**Supplementary Information:**

The online version contains supplementary material available at 10.1038/s41598-026-44137-9.

## Introduction

Empathy can be defined as “the drive or ability to attribute mental states to another person/animal, and entails an appropriate affective response in the observer to the other person’s mental state”^1^. It is widely acknowledged that empathy consists of two components: (a) emotional empathy and (b) cognitive empathy. The latter “denotes the ability to take the mental perspective of others, allowing one to make inferences about their mental or emotional states”^2^. The former reflects “the tendency to vicariously experience other individuals’ emotional states”^3^ and involves somatosensory and motor representation of another’s state^4^.

Besides this influential differentiation, cognitive and emotional empathy can be further divided into state and trait empathy^5^. As some authors suggest, the difference between state and trait empathy corresponds to the difference between competence and achievement^6^. Whereas competence refers to the ability, achievement reflects dispositional drive. Thus, state empathy reflects the upper level of possible empathy, whereas trait empathy denotes the tendency to experience the emotions of others^6^.

Regarding empathy as a state, several studies examined its neural basis. For example, the meta-analysis of Lamm et al.^7^ indicated that during emotional empathy tasks, bilateral anterior insula and medial/anterior cingulate were consistently activated across analysed functional Magnetic Resonance Imaging (fMRI) studies. However, as Uribe et al.^8^ note, only a handful of studies exist that examined the neuroanatomical basis of empathy as a trait. More importantly, these studies suffer from methodological limitations in their design that question the validity of their results. The first of these methodological limitations is the use of certain empathy measures, the validity of which was questioned by a number of studies. For instance, the Voxel-Based Morphometry (VBM) studies of Banissy et al.^9^ and Michaels et al.^10^ used the Interpersonal Reactivity Index - IRI -^11,12^ for measuring empathy, which assesses four dimensions of empathy. Although this self-report measure is the most widely used assessment tool for measuring empathy^13,14^, some of its subscales have been criticized for potentially capturing self-control, imagination^15^, and neuroticism^16^.

Along with the problematic measures of empathy, structural neuroimaging research on empathy has often relied on Voxel-Based Morphometry (VBM). However, VBM is susceptible to partial volume effects, where geographically close but functionally distinct areas are conflated, hindering accurate cortical assessment^17^. Furthermore, VBM lacks the sensitivity to discern subtle cortical features. These methodological problems can be bridged by Surface-Based Morphometry (SBM)^18^, which offers a precise reconstruction of the cortical surface and allows for the extraction of specific features such as gyrification and sulcal depth.

In contrast to VBM, SBM is not prone to partial volume effects and allows the extraction of more detailed information about cortical structure, such as gyrification, sulcal depth, or cortical thickness. Sulcal depth, in particular, provides a measure of cortical folding that is less susceptible to measurement errors than cortical thickness and may reflect early neurodevelopmental processes^19,20^. Despite these advantages of SBM, to the best of our knowledge, there are only two structural studies on trait emotional empathy^21,22^ that used the SBM technique. However, these studies have two notable limitations. First, these studies did not investigate whether the brain regions consistently activated in fMRI studies of empathy display structural associations with trait empathy when examined using SBM. More specifically, previous functional neuroimaging studies^7,23^ suggested that neural activity in the bilateral insula and the bilateral anterior cingulate cortex (ACC) is associated with state empathy, alongside other key regions such as the Inferior Parietal Lobule (IPL), Superior Temporal Sulcus (STS), Dorsolateral Prefrontal Cortex (DLPFC), Orbitofrontal Cortex (OFC), and Inferior Frontal Gyrus (IFG). However, the two SBM studies did not test research hypotheses concerning these regions.

The second limitation is that these two studies only explored the relationship between empathy and cortical thickness but omitted other cortical features that might be important for the explanation of individual differences in empathy and for empathy prediction. Currently, there is no study that explores associations between trait emotional empathy and either gyrification or sulcal depth of cortical regions.

Thus, to fill this knowledge gap, our study had two aims. First, to verify research hypotheses grounded in the assumption that the neural mechanisms underlying the momentary experience of empathy (state) are intrinsically linked to the stable disposition to experience empathy (trait). Specifically, we posited that the structural properties of brain regions consistently recruited during state empathy tasks—specifically the bilateral insula and dACC—would predict individual differences in trait emotional empathy. Consequently, we hypothesized that cortical thickness in the left insula (Hypothesis 1), right insula (Hypothesis 2), and left dACC (Hypothesis 3) is positively associated with trait emotional empathy. Second, to explore the predictive accuracy of gyrification, sulcal depth, and cortical thickness for trait emotional empathy.

## Methods

### Participants and assessment tools

The study recruited adults from the Czech Republic, all over the age of 18. The study subjects were gathered using a convenience sampling approach from February 2019 to September 2020. These individuals participated in an online survey developed at the Olomouc University Social Health Institute (OUSHI) as a segment of a project focusing on the relationship between spirituality and health. In addition to the survey, the study required participants to undergo magnetic resonance imaging (MRI).

Initially, our sample size consisted of 76 participants. However, screening of the missing values indicated that behavioral data from 4 (5%) respondents were missing. Consequently, these respondents were removed from the dataset listwise (*n* = 72). Quality assessment results indicated that no respondents completed the questionnaire in an excessively short time (defined as duration < 3 min). Outlier screening indicated that 1 respondent answered almost all questions/items in the online survey in the same way. Therefore, this respondent was removed from the analysis (*n* = 71). We also removed respondents with missing MRI data (*n* = 7; remaining *n* = 64). Finally, the MRI images, specifically T1 weighted scans, underwent a visual quality check. 2 scans were either missing or discarded due to quality issues, such as missing sections of the frontal lobe. This resulted in a final number of participants: 62 (Age: *M* = 30.55, range 18–85, *SD* = 12.02; Females: 56.45%).

### Measures

#### Toronto empathy questionnaire (TEQ)

To assess the degree of empathy, the study used the Toronto Empathy Questionnaire (TEQ). This scale is recognised as a valid and reliable tool for assessing trait empathy with a focus on its emotional aspect^16^. The Czech adaptation of the scale^24^ consists of 7 questions, each rated on a 5-point Likert scale where ‘0’ refers to “Never” and ‘4’ refers to “Always”. A greater sum score on the TEQ indicates higher empathy. In this study, Cronbach’s α was 0.73, 95% CI[0.61–0.82] and McDonald’s ω: 0.82. We chose the TEQ because alternative scales, such as the IRI, have been criticized for potentially capturing related constructs such as self-control, imagination^15^, and neuroticism^16^.

The sociodemographic characteristics of participants were obtained through an online questionnaire.

### Acquisition and preprocessing of MRI data

MRI imaging used a Siemens Prisma 3 Tesla MR scanner (Siemens Medical, Erlangen, Germany) with a 64-channel head-neck coil at the Multimodal and Functional Imaging Laboratory (MAFIL) at the Central European Institute of Technology (CEITEC), Masaryk University in Brno, Czech Republic. For capturing structural brain images, a T1-weighted Magnetization Prepared Rapid Gradient Echo (MPRAGE) sequence was employed, with the following parameters: Repetition Time (TR) of 2300 ms, Echo Time (TE) of 2.33 ms, Inversion Time (TI) of 900 ms, an acquisition matrix of 224 × 224, 240 sagittal slices, a voxel dimension of 1 × 1 × 1 mm, Parallel Acquisition Technique factor (PAT) factor of 2, and a Flip angle of 8°.

The MRI scans were analysed using the Statistical Parametric Mapping (SPM) 12 (Wellcome Department of Imaging Neuroscience Group, London, UK) on MATLAB 2018 software (The MathWorks, Natick, MA, USA). Initial image processing involved segmenting into cerebrospinal fluid, white matter, and gray matter. Subsequently, images were aligned to the Montreal Neurological Institute (MNI) space. We conducted a visual examination to identify any artifacts in the images, such as motion or gross anatomical artifacts. Moreover, we explored the homogeneity of the sample by two methods: first, by average correlation (assessing the homogeneity of the images), and second, by weighted overall image quality (which incorporates spatial resolution and noise).

### Surface-based morphometry and ROI analysis

The SBM was employed to measure cortical thickness, surface folding, and sulcal depth using the Computational Anatomy Toolbox (CAT12) software^18^. The cortical thickness images underwent smoothing with a 15 mm Gaussian kernel, and the gyrification index and sulcal depth were smoothed with a 20 mm kernel Full Width at Half Maximum - FWHM - ^18,25^. The pipeline from the projection-based thickness method^18^ was used to assess the cortical thickness and reconstruct the central cortical surface in a single process. The local gyrification index was derived from the absolute mean curvature^25^. The sulcus depth was calculated as the Euclidean distance between the central surface and the convex hull^26^. We used these resampled and smoothed GI maps for the whole-cortex Surface-Based Morphometry (SBM) analyses of gyrification described later in the “Statistical analysis” section.

The Regions of Interest (ROI) analysis focused on the two structures: bilateral insula (left: x = -42, y = 18, z = 0; right: x = 38, y = 24, z = -2) and the left dorsal Anterior Cingulate Cortex (dACC) (x = -2, y = 24, z = 38). Coordinates of these two MNI locations were acquired from the meta-analysis of Fan et al.^23^, which showed that neural activity in these two structures was positively associated with state empathy. We acknowledge that, based on some parcellation schemes, the ACC coordinates may fall within the mid-cingulate cortex (MCC). We used Schaefer et al.^27^ cortical atlas with 600 parcellations to estimate mean cortical thickness, gyrification, and sulcal depth values in individual brain areas. In the next step, we mapped MNI coordinates onto the Schaefer et al.^27^ atlas template to obtain the label of cortical surface areas from the Schaefer atlas for ROI analysis. We also acknowledge that the left insula coordinate is located in close proximity to the Inferior Frontal Gyrus (IFG). A more detailed description of this process is depicted in Supplementary Material 1.

### Statistical analysis

In the first step, we checked abnormalities in TEQ score using the Median Absolute Deviation (MAD) method and found 3 outliers. However, checking these outlying values did not suggest that study subjects would respond to questionnaire items in the same way. For this reason, these subjects were not excluded from the dataset. Although the Shapiro-Wilk test indicated that TEQ scores are not normally distributed (*W* = 0.96, *p* = 0.030), visual inspection of the histogram and Q-Q plot suggested that TEQ data are not highly skewed or kurtotic. For this reason, we used parametric methods to test the hypotheses of this study.

Next, we compared sociodemographic groups in their empathy level. Due to the heterogeneity of variances across certain sociodemographic groups, we used robust versions of parametric tests for group comparisons. For the ROI analysis, correction for multiple comparisons was not applied as these tests represented a limited number of specific, a priori hypotheses derived from previous meta-analyses^23^. Next, our analysis focused on hypothesis testing. These hypotheses were predicated on the assumption that the structural integrity of brain regions consistently recruited during functional state empathy tasks—specifically the bilateral insula and dACC^7,23^—would predict individual differences in trait emotional empathy. The Linear Mixed Effect (LME) models were used to test the study hypotheses. We fitted three LME models, each time with the same dependent variable (TEQ score) but with differing independent variables. These independent variables were cortical thickness in (1) left insula, (2) right insula, and (3) left dACC. Adjusting variables (age and gender) were the same across all LME models. The definition of the LME model is depicted in the equation below.

$$\begin{gathered} {\mathrm{TE}}{{\mathrm{Q}}_i}\sim N\left( {{\alpha _{j\left[ i \right]}},{\sigma ^2}} \right) \hfill \\ {\alpha _j}\sim N\left( {\gamma _{0}^{\alpha }+\gamma _{1}^{\alpha }\left( {{\mathrm{thickness}}} \right)+\gamma _{2}^{\alpha }\left( {{\mathrm{age}}} \right)+\gamma _{3}^{\alpha }\left( {{\mathrm{Gende}}{{\mathrm{r}}_{{\mathrm{Female}}}}} \right),\sigma _{{{\alpha _j}}}^{2}} \right){\mathrm{,~for~subject\_id~j~=~1,}} \ldots {\mathrm{,J}} \hfill \\ \end{gathered}$$

In this equation, ${\mathrm{TEQ}}i$ is the dependent variable, while $\alpha j\left[ i \right]$ represents individual-specific intercepts. $\gamma _{0}^{\alpha }$ denotes the baseline, $\gamma _{1}^{\alpha }$ the effect of cortical thickness, $\gamma _{2}^{\alpha }$ the effect of age, and $\gamma _{3}^{\alpha }$ the effect of female gender. These parameters are applied across subjects, indexed by *j* from 1 to *J*. Checking of statistical assumptions of the LME models suggested that there might not be linear relationships between cortical thickness and trait emotional empathy. For this reason, after testing the hypotheses using a linear model, we fitted non-linear mixed effect models containing (1) quadratic, (2) cubic, and (3) quartic terms. Consequently, these models were compared using the Bayesian Information Criterion (BIC) to select the best-fitting model.

## Predicting degree of empathy from cortical measures: Machine Learning (ML) analysis using Sparse Partial Least Squares regression (SPLSR)

In a further step, we aimed to predict empathy scores from morphometric measures such as sulcus depth, gyrification, and cortical thickness. In more detail, we used the Schaefer cortical atlas to extract mean values of sulcus depth, gyrification, and cortical thickness from each cortical surface area. Next, we used these values to predict the degree of empathy. To predict the degree of empathy from morphometric measures, we employed Sparse Partial Least Squares Regression (SPLSR), which is suitable for handling the multicollinearity that is often found in morphometric measures^28–30^. Generally, the SPLSR (Sparse Partial Least Squares Regression) is an extension of the older method (i.e., Partial Least Squares Regression - PLSR), which is a multivariate method with many applications in electrophysiology and neuroimaging research^31^. The main principles behind both SPLSR and PLSR are the same: the decomposition of independent variables to create uncorrelated latent variables that yield maximum covariance with an outcome variable^28^. The degree of association between these independent variables and latent variables is called component loading^32^. After latent variables are created, they can be regressed on the outcome. The degree of association between these latent variables and the outcome variable can be represented by regression coefficients^33^.

However, unlike PLSR, SPLSR combines variable selection and modelling in a single step^30,34^. In the SPLSR, the Least Absolute Shrinkage and Selection Operator (LASSO) is used to penalize loading vectors to latent variables during their construction so that only important features can be selected^34^. Taken together, the key difference between PLSR and SPLSR is that SPLSR uses only the most important features while omitting those that are less influential^34^.

### Preprocessing and model selection

Prior to fitting SPLSR models, variables with zero or near-zero variance were removed. Subsequently, data were centered and scaled to ensure equal weighting of features in the sparse selection process. While our morphometric measures (thickness, depth, gyrification) were analyzed in separate models and share measurement scales within each model, standardization was necessary because (1) different brain regions exhibit heterogeneous variance even within the same measure type, and (2) the LASSO penalty in SPLSR is sensitive to feature scale—without standardization, feature selection would be biased toward high-variance regions rather than truly informative ones. Standardization was performed independently within each cross-validation fold using parameters derived from training data only.

To select the optimal number of components, we implemented a nested cross-validation procedure. We tested components ranging from 1 to 61 (the number of observations – 1) and used the Bayesian Information Criterion (BIC) to identify the optimal number. For each candidate number of components ($k$), we employed a Nested Leave-One-Out Cross-Validation (Nested LOO-CV) framework: (1) the outer loop performed LOO-CV where each of the 62 participants was iteratively held out as a test set; (2) for the remaining $n-1$ training participants, an inner LOO-CV loop tuned the keepX parameter (number of selected features, tested at intervals of 5 from 5 to total number of features (i.e., 600); (3) the cross-validated prediction error from the outer loop was then used to calculate $BIC = n \times log\left( {RSS/n} \right) + k \times log\left( n \right)$, where RSS is the residual sum of squares from cross-validation; (4) the number of components yielding the lowest BIC was selected for final model building.

### Final model evaluation

After determining the optimal number of components for each morphometric measure, we evaluated the final SPLSR models (one each for thickness, depth, and gyrification) using Nested LOO-CV. The outer loop (*n* = 62 iterations) estimated generalization performance, while the inner loop tuned keepX for each training fold using the same parameter search space described above. Crucially, all preprocessing steps (removal of zero/near-zero variance features, centering, and scaling) were performed independently within each fold of the cross-validation loop, using parameters derived solely from the training set to transform the corresponding test set. This ensures that performance estimates are unbiased and that no information leakage occurs from test data into model training.

### Performance metrics and significance testing

Model performance was assessed using three key statistics: (1) the Root Mean Square Error of Prediction (RMSEP = $$\sqrt {\sum {{\left( {{y_{obs}} - {y_{pred}}} \right)}^2}/n}$$), quantifying average prediction error; (2) the cross-validated coefficient of determination $$\left({Q^2}=1 - \sum {\left( {{y_{obs}} - {y_{pred}}} \right)^2}/\sum {\left( {{y_{obs}} - \overset{\lower0.5em\hbox{$\smash{\scriptscriptstyle\leftharpoonup}$}} {y} } \right)^2}\right)$$, reflecting the proportion of variance in empathy scores predictable from unseen data, where negative values indicate worse-than-baseline prediction; and (3) the coefficient of determination ($${R^2}=cor{\left( {{y_{obs}},{y_{pred}}} \right)^2}$$), representing the squared correlation between observed and cross-validated predicted values. To assess statistical significance, we performed permutation testing with 1000 iterations: in each iteration, TEQ scores were randomly shuffled hyperparameters retuned, and models re-evaluated using LOO-CV.

Finally, based on results from the final SPLSR model, we conducted variable importance analysis to select cortical areas with the highest regression coefficients in relation to the empathy score. The R statistical software (version 4.3.0;^35^ and Python 3^36^ were used for most statistical analyses. In the R statistical environment, the following packages were primarily used: the mixOmics^37^ for SPLSR analyses, the psychtoolbox^38^ for descriptive analysis, and demographic group comparison. For a complete list of used packages, including their versions, see online supplementary materials on the Open Science Framework (10.17605/OSF.IO/5ZC47).

## Results

Socio-demographic testing indicated that most participants had a university education, were not married, were female, and were religious. The Wilcoxon test indicated that there was a significant difference between males and females in empathy scores with medium effect size. Gender was subsequently included as a covariate in all regression models (except SPLSR) to control for this potential confound. Further details on socio-demographic differences can be found in Table 1.

**Table 1:** Socio-demographic characteristics of participants.

variable	*n*(%)	TEQ group difference	TEQ M(SD)
Gender			
1. Male	27(43.55)	W = 256, *p* = 0.002, rbc = 0.39	20.67 (2.86)
2. Female	35(56.45)		22.86 (2.83)
Education			
1. High school or lower	22(35.48)	W = 364, *p* = 0.264, rbc = 0.14	21.41 (2.7)
2. University	40(64.52)		22.18 (3.19)
Family status			
1. Not married	47(75.81)	W = 328.5, *p* = 0.697, rbc = 0.05	21.77 (3.17)
2. Married	15(24.19)		22.33 (2.55)

Note. TEQ = Toronto Empathy Questionnaire, M = mean, SD = Standard Deviation, n = number of participants, rbc = rank-biserial correlation (effect size for Mann-Whitney U test).

### Regions of Interest analysis: association between cortical thickness and empathy

**Fig. 1:**
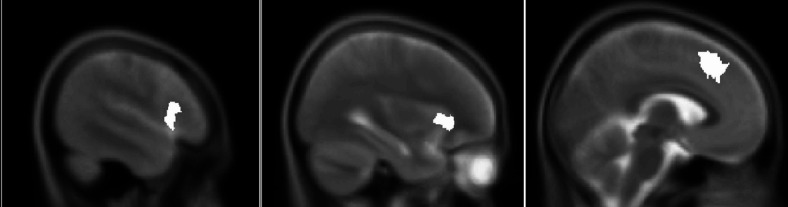
From left - neural localization of the left insula, the right insula and the left ACC.

Linear mixed effect models showed that contrary to our Hypothesis (H1), there was a significant negative association between the cortical thickness of the left insula and empathy score: B = -4.72; 95% CI [-8.52, -0.92]; SE = 1.96; *p* = 0.019, meaning that lower cortical thickness was associated with higher trait emotional empathy. In contrast with our second hypothesis (H2), there was no significant association between cortical thickness in the right insula and trait emotional empathy: B = -0.64; 95% CI [-4.97, 3.70]; SE = 2.17; *p* = 0.770. Finally, contrary to our third hypothesis (H3), there was a significant negative association between cortical thickness in the left dACC and empathy: B = -5.47; 95% CI [-9.48, -1.46]; SE = 2; *p* = 0.008. We also verified these results using non-parametric Spearman correlations, which confirmed the direction and significance of the reported associations (see correlation table in Supplementary Material 2). The neural localisation of these areas is depicted in Fig. 1 below.

**Fig. 2:**
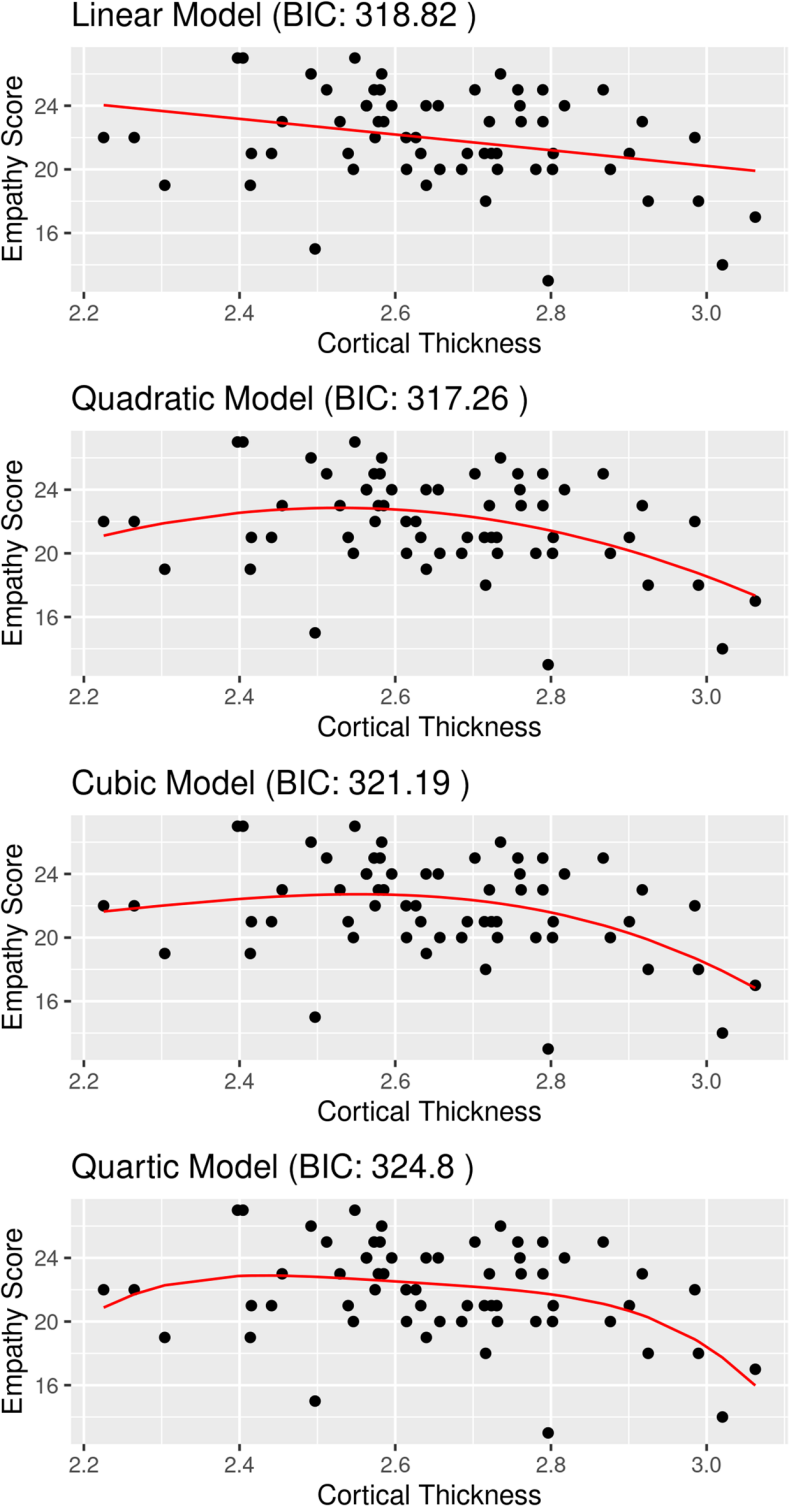
Fit of different model terms linear, quadratic, cubic, quartic in the left insula, BIC = Bayesian Information Criterion.

In a further step, we tested relationships between the cortical thickness of these three areas and trait emotional empathy using non-linear statistical models. Results revealed that in the left dACC, non-linear models did not outperform the linear model in predicting trait emotional empathy based on cortical thickness (see Supplementary Material 2). However, in the left insula, the quadratic model outperformed the linear model in predicting the degree of trait emotional empathy (Fig. 2 and Supplementary Material 2). In the same neural area, more complex models such as cubic or quartic did not predict the degree of trait emotional empathy well (Fig. 2). In the right insula, no significant effect was found in any of the non-linear models (Supplementary Material 2).

### Predicting the degree of empathy using SPLSR

We trained SPLSR models to predict TEQ scores from whole-cortex features (Schaefer atlas parcellation), separately for cortical thickness, sulcal depth, and gyrification. BIC-based component selection identified 1 component(s) for depth, 1 for gyrification, and 1 for thickness (Supplementary Fig. 1 in Supplementary Material 2). These values were used to fit the respective SPLSR models.

Although in-sample fit appeared moderate for some models (e.g., fitted ${R^2}$ of 59.50% for gyrification, 30.53% sulcal depth, and 30.50% cortical thickness), out-of-sample performance under leave-one-out cross-validation was poor. Cross-validated predictive performance was worse than a mean-only baseline, with negative ${Q^2}$ values for all models (gyrification ${Q^2}= - 0.35$; sulcal depth ${Q^2}= - 0.14$; cortical thickness ${Q^2}= - 0.18$). Consistent with this, prediction error (RMSEP) was 3.20 for sulcal depth, 3.48 for gyrification, and 3.25 for cortical thickness.

**Fig. 3:**
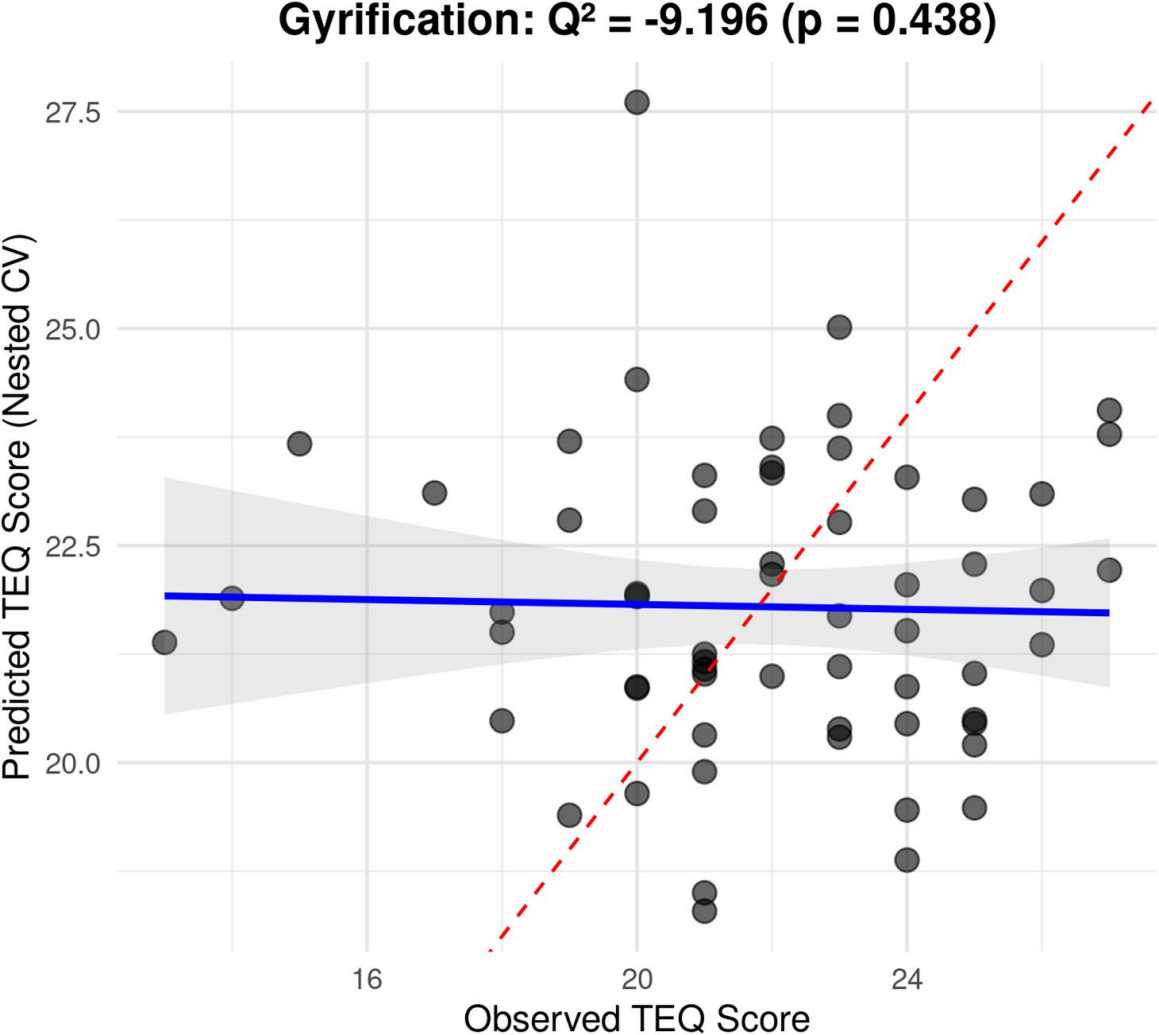
Relationship between predicted values (y-axis) by the model and observed values (x-axis) in TEQ score using gyrification for prediction of empathy score. The blue line shows the linear relationship between observed and predicted values with 95% confidence interval (gray shading). The red dashed line indicates perfect prediction (y = x). Q^2^ = Cross-validated coefficient of determination (predictive accuracy metric), p = p-value from permutation test (1000 permutations), CV = Cross-validation (leave-one-out method).

Given that ${Q^2}<0$ indicates lack of useful generalization, we do not interpret the fitted SPLSR regression coefficients in the main text. Coefficients are therefore provided only as descriptive material in Supplementary Material 2. Figure 3 depicts the relationship between predicted and observed empathy scores for the gyrification model.

## Discussion

The aim of this study was to test research hypotheses derived from functional studies and to test the predictive accuracy of gyrification, sulcal depth, and cortical thickness for trait emotional empathy. We found a negative association between the cortical thickness of the left insula, dACC, and trait emotional empathy. In addition, the relationship between cortical thickness in the left insula and trait emotional empathy was non-linear. In contrast, right insula thickness was unrelated to trait emotional empathy in both linear and non-linear models. Regarding the machine learning analysis, while the predictive accuracy of cortical features was moderate on the training data, the cross-validated performance on out-of-sample data was poor. Thus, morphometry alone did not support useful individual-level prediction of empathy score in this sample.

Our results showed a negative association between the cortical thickness of the left insula and left dACC with trait emotional empathy, which contrasts with prior SBM research suggesting either a positive relationship^39^ or no significant relationship^22^. The discrepancy between our study and that of Massey et al.^39^ could be explained by the fact that Massey et al. focused on the cognitive aspect of empathy, whereas the present study examined trait emotional empathy. In the context of Schmidt et al.^22^, the inconsistency of results could be explained by a small sample size. As Schmidt et al.^22^ included only 20 healthy subjects, it is possible that with a larger sample size, Schmidt et al.^22^ would have found a negative link between left insula, dACC, and trait emotional empathy, and thus, their results would be in line with the present study findings.

Importantly, it remains to be explained why our results do not correspond with our hypotheses (H1 and H3), as well as the results from functional studies, which found a positive link between neural activity in the left insula, dACC, and empathy. While our ROI selection was predicated on the hypothesis that state and trait emotional empathy share a common neural basis, our results suggest that the nature of this association differs between modalities. One possible explanation could be that the present study focused on empathy as a trait, whereas functional studies focused on empathy as a state^40,41^. Specifically, efficient neural processing in these shared regions—manifesting as high activation during state empathy—might be structurally supported by cortical pruning (thinner cortex) in trait emotional empathy. This explanation can also be supported by the results of Banissy et al.^9^, who examined trait emotional empathy and also found a negative relationship between trait emotional empathy and grey matter density in the left insula and dACC. However, we interpret this distinction with caution. Methodological heterogeneity, such as the use of different measurement tools (e.g., TEQ vs. IRI) or variations in sample size and statistical power across studies, may also contribute to the observed discrepancies.

In contrast with our hypothesis (H2), we did not observe a significant relationship between the cortical thickness in the right insula and trait emotional empathy. This contradicts the results of Eres et al.^42^ but is consistent with the findings of Banissy et al.^9^. One reason for this discrepancy could be statistical power. Specifically, Eres et al.^42^ found a small correlation between the right insula and trait emotional empathy (*r* = 0.23) using 176 participants. Thus, it is possible that neither our study (*n* = 62) nor Banissy et al.^9^, with 118 participants, possessed sufficient statistical power to detect such a small effect, compared with the adequately powered study of Eres et al.^42^. However, beyond statistical power, fundamental methodological differences likely play a significant role. For instance, Eres et al.^42^ utilized VBM, which is susceptible to partial volume effects, whereas our study employed SBM, yielding potentially different structural quantifications. Furthermore, variability in the empathy measures used across studies (e.g., IRI versus TEQ) may target slightly different facets of the construct, complicating direct comparison. This explanation aligns with the results of the SBM study of Schmidt et al.^22^, which also did not find an association between the right insula and trait emotional empathy using 20 subjects. Another explanation is that in the study of Eres et al.^42^, the center of grey matter density was anatomically located on the border between the right insula and Inferior Frontal Gyrus (IFG). Thus, it is possible that in the study of Eres et al.^42^, the primary source of an effect comes from the IFG rather than from the right insula. This explanation seems to be supported by the studies causally linking the IFG with emotional empathy^26^.

Our analysis also suggested a non-linear relationship between cortical thickness in the left insula and trait emotional empathy. This finding is difficult to compare with previous studies since, to the best of our knowledge, no study has examined the relationship between neural structure and trait emotional empathy using non-linear methods. Although non-linear modeling is rarely used to study the relationship between brain structure and trait emotional empathy, it is not novel in other fields of neuroscience. For instance, other studies, such as those by Bohon and Welch^43^ and Corriveau-Lecavalier et al.^44^, have used similar methods to explore different brain-body relationships. For example, Bohon and Welch^43^ showed that Body Mass Index (BMI) has a two-phase relationship with hippocampal size: initially, BMI increase is related to an increase in grey matter volume in the hippocampus; however, beyond a certain point, further increases in BMI were related to decreased hippocampal volume. In a similar way, we hypothesize that a certain level of thickness in the left insula is beneficial for trait emotional empathy, but beyond that level, the relationship changes. However, we emphasize that this interpretation implies a theoretical mechanism that extends beyond our current investigation. Given the exploratory nature of our non-linear analysis, these findings should be considered preliminary and require replication in larger, independent samples to determine their stability.

Leave-one-out cross-validation revealed poor performance of all cortical features in predicting the degree of trait emotional empathy in unseen data. This pattern is broadly consistent with prior work on socio-affective traits, where reported brain–trait associations are often small and heterogeneous^45–47^. High-dimensional morphometric models also frequently show limited generalization unless sample sizes are large and/or predictors are multimodal^48–50^. Several factors may explain why predictions failed to generalize. First, the high dimensionality of parcel-wise predictors relative to sample size leads to unstable parameter estimates and poor generalization performance^47,50^. Second, measurement noise and the broad conceptual scope of self-report trait measures impose fundamental limits on prediction accuracy^51^. Third, trait emotional empathy likely depends on distributed, network-level and context-dependent processes that morphometry alone cannot adequately capture^23,52–54^.

Importantly, the lack of out-of-sample prediction does not contradict the ROI findings. ROI analyses address whether targeted regions relates with TEQ at the group level, whereas prediction demands effects that are sufficiently large and stable to support accurate inference for new individuals. Thus, it is plausible for small, regionally specific associations to be present while the aggregate morphometric pattern remains insufficient for reliable person-level prediction.

### Strengths and limitations

This study has several strengths. First, advanced neuroimaging analysis techniques such as SBM were used to explore less-studied structural correlates of trait emotional empathy, like sulcus depth. This allowed us to examine less-studied morphometric features (e.g., sulcal depth). Second, for assessing trait emotional empathy, we used a measurement tool that is valid and reliable. This, in turn, increases the likelihood that our findings could be more generalisable and replicable by future studies. Third, this study primarily relied on MNI coordinates instead of neuroanatomical labels to evaluate research hypotheses using ROI. This decreases the chance that we accidentally focus on a different region than intended due to differences in neuroanatomical labeling.

Our study also has some limitations. First, we used cross-sectional data, which precludes causal inferences. Second, our sample size (*n* = 62) was modest relative to the number of features, which increases variance in model estimates and raises the risk of overfitting. SPLSR provided a sparse model representation, but the poor cross-validated performance indicates limited generalizability; therefore, regression coefficients should be treated as exploratory and interpreted with great caution. With only 62 participants and several hundred cortical parcels, the sample-to-feature ratio fundamentally constrains what the machine learning analysis can demonstrate; the negative cross-validated ${Q}^{2}$ values are, in this light, an expected consequence of insufficient statistical power to recover stable multivariate patterns. Overall, sample size remains the largest limitation of the present study, and our findings thus need to be interpreted with great caution.

### Implications for practice

At this stage, the practical implications of our findings are primarily research-oriented rather than clinical. Although neuroimaging-based models of socio-emotional traits are sometimes discussed as promising assessment tools of the future, the present results do not support individual-level inference: whole-brain morphometry did not generalize under out-of-sample evaluation. Any clinical application would require strong external validation across cohorts (ideally across sites, scanners, and acquisition protocols) to demonstrate stable generalization across populations and measurement conditions.

### Implications for research

There are four main implications for future research. First, research focusing on individual-level prediction of empathy score should treat out-of-sample performance as the primary criterion for model usefulness. Second, future studies should test whether white-matter organization within the insula relates to trait emotional empathy. Third, future work on neural bases of empathy should continue to examine morphometric measures that are less frequently used in social neuroscience—such as sulcal depth. Doing so may expand current models of the neuroanatomical basis of socio-emotional functioning and clarify which aspects of cortical morphology are most reproducibly associated with individual differences. Finally, transparently reporting null predictive results is itself informative for the field. Publication bias toward positive findings can distort the literature and inflate expectations about the feasibility of individual-level brain–trait prediction. By applying rigorous nested cross-validation and openly reporting the negative outcomes, the present study helps calibrate realistic expectations about what small-sample morphometric machine learning can achieve for socio-affective traits and may guide future researchers toward appropriately powered designs.

## Conclusion

Our study provided evidence that using non-linear models might offer additional insights into complex relationships between cortical morphology and trait emotional empathy. However, the machine learning analyses did not yield reliable out-of-sample predictions, and this limitation is most likely attributable to the small sample size (*n* = 62) relative to the high-dimensional feature space. Consequently, the predictive findings of this study should be considered exploratory, and any conclusions regarding the capacity of cortical morphometry to predict trait emotional empathy at the individual level must await replication in substantially larger and independent samples. Future researchers could explore the connection between trait emotional empathy and white matter to gain a more comprehensive understanding of the neural basis of this construct.

## Supplementary Information

Below is the link to the electronic supplementary material.

Supplementary Material 1

## Data Availability

Anonymised data, code, trained ML model, and other materials related to this study are available at the Open Science Framework (OSF) website under the following DOI: [https://doi.org/10.17605/OSF.IO/5ZC47](https:/doi.org/10.17605/OSF.IO/5ZC47).
