# Supplementary material for: Predicting the degree of trait emotional empathy from cortical features using surface-based morphometry
Source: Sci Rep. 2026 Mar 25;16:14893. doi: 10.1038/s41598-026-44137-9 (PMC13168272; doi:10.1038/s41598-026-44137-9)
Supplement: Supplementary file 1 — Supplementary Material 1 [file 41598_2026_44137_MOESM1_ESM.zip › Supplementary_material_1.html]

README


# Supplementary material 1

## Table of Contents

1. Reproducibility
2. Preparation of
   neuroimaging data and processing
3. Regions
   of Interest Analysis (ROI) - detailed description of the
   process
4. Labeling
   neural regions in Exploratory Analyses

# Reproducibility

If you want to reproduce the study results using aggregated MRI data,
you need to follow these steps:

1. Download the entire directory of the project from the OSF project
   website.
2. Download and install Docker
   Desktop.
3. Open the terminal in the project directory on your local machine
   and type `docker compose up --build`. After the Docker image
   is built, open the following link.
4. After the RStudio server opens, all you need to do is open the
   following Rmd script: mri\_std\_prepar\_script.Rmd and press
   the `knit` button. After that, you will get the output in
   `docx` format containing the results of all the main analyses
   of the study.

# Preparation of neuroimaging data and processing

This study combines R code with Matlab code in a single processing
pipeline. Thus, if you want to reproduce the presented study results
from scratch (i.e., using raw MRI data, which is not necessary to fully
reproduce the study reults), the following steps are needed.

1. Download `MRI_data` folder from google drive and unzip
   them in Data directiry. Than the following steps
   needs to be conducted:

   1. open mri.std.prepar.script.Rmd and run
      all code until the chunk named: `Extraction_of_MRI_data`.
      After running this chunk, you should have MRI data extracted in Data directory. It is necessary that sublolder of
      `MRI_data` consists of folders of individual subjects
      (e.g. 3458B, 2879B etc) and not another subfolder called
      `MRI_data`. Running code until this chunk will also select
      codes of respondents having some data in TEQ and age variables and save
      them for futher processing in MATLAB.
2. In the next step, run `Segment_smooth_normalization_2`
   script, which can be found in the Matlab\_scripts directory. This script will do
   for you:

   1. Segmentaion - Splits gray matter (GM), white matter (WM) and
      cerebrospinal fluid (CSF).
   2. Extraction of Additional surface parameters (sulcus depth and
      gyrification)
   3. Resampling and smoothing of GYRIFICATION (23 mm filter used for
      smoothing)
   4. Resampling and smoothing of SULCUS DEPTH (12 mm filter used for
      smoothing)
3. Quality control of images

   1. In order to perform the first step of image quliaty control, there
      is need to examine individual image qulity using pdf files resulting
      from the previous step. This can be done by running chunked named
      `quality_control_of_mages` in
      `mri.std.prepar.script.Rmd` file.

4. Regression analysis this step has the primary aim of a quality
   check:
   - 1. it consists of definition of independent variables and extracts data
        from text files consisting of values of these independat variables.
   - 2. Consists of quality check of images.
     - after the last low quality respondents are identified, they must be
       excluded in `mri.std.prepar.script.Rmd`. In this R script,
       there is need to add codes of subject that we want to remove. These
       codes should be added to **every** relevant filter function
       excluding respondents in Rmd file
       e.g. `filter(!Kod.fMRI1 %in% "3995B" & !Kod.fMRI1 %in% "4045B" & !Kod.fMRI1 %in% "3993B" & !Kod.fMRI1 %in% "4000B" & !Kod.fMRI1 %in% "3429B" & !Kod.fMRI1 %in% "3175B")`
     - after these respondents are filtered out, there is need to knit
       `mri.std.prepar.script.Rmd` producing updated txt files files
       with filered codes
     - go back to `smoothing_of_VBM_data_and_TIV_estimate.m`
       script and rerun the code. Than, TIV values are estimated for reduced
       sampe.
   - 3. after finish quality checking the regression analysis code in
        `Regression_quality_check_VBM.m` can be rurunned. Note that
        the step `b)`and `c)` can be repeated multiple
        simes untill all images of problematic subjects are removed.

# Regions of Interest Analysis (ROI) - detailed description of the process

- It was critical to test our hypotheses in regions as similar as
  possible to those found in past research.
- For this reason, the following steps took place:

  1. We extracted MNI coordinates from previous
     studies examining the neural bases of empathy (bilateral insula
     (left: x = -42, y = 18, z = 0; right: x = 38, y = 24, z = -2) and the
     left ACC (x = -2, y = 24, z = 38)). These coordinates were derived from
     meta-analytic data (Fan et al., 2011) which labeled this region as ACC,
     although we acknowledge that based on some parcellation schemes, this
     may fall within the mid-cingulate cortex.
  2. Next, using the SPM12 Display function, we opened the Schaefer 2018
     600 parcels atlas and wrote MNI coordinates in the mm window. After
     that, intensity values were displayed (see Figure below):
  3. In the next step, we extracted the intensity value displayed in the
     figure above.
  4. Consequently, the XML
     file containing a list of intensities was opened.
  5. In this XML
     file, we found the corresponding intensity (e.g., 281) and also the
     corresponding name of the region in the Schaefer atlas (e.g.,
     `17Networks_LH_DefaultB_PFCv_7`).
  6. Consequently, cortical measures of this anatomical region (e.g.,
     `17Networks_LH_DefaultB_PFCv_7`) were used in further ROI
     analyses.

  This approach provided a relative certainty that the analyzed region
  of interest (e.g., `17Networks_LH_DefaultB_PFCv_7`) in fact
  represents MNI coordinates from the previous
  studies examining the neural bases of empathy.

# Labeling neural regions in Exploratory Analyses

## Overview

The main aim of this procedure was to translate Schaffer atlas into
Automatic Anatomical Labelling 3 (AAL3). To reach this goal, there is
need to run two chunks of Python code in mri.std.prepar.script.Rmd
extracting appropriate neuroanatomical labels based on intensities from
XML files and mapping these intensities to MNI coordinates using NIfTI
files.

## Table of Contents

1. Extraction
   and Saving of Intensities from XML File
2. Saving MNI
   Coordinates with Intensities

## Extraction and Saving of Intensities from XML File

- In the first step, we copied the Schaefer 2018 atlas as an XML
  file from the CAT12 toolbox directory in SPM to the present
  directory.
- Next, we extracted the following information from this XML
  file:
  - Intensities
  - Anatomical names from the Schaefer Atlas.Next, these pieces of information were stored in a CSV file for further
  processing.

## Saving MNI Coordinates with Intensities

- In the next step, we also loaded a NIfTI
  image file containing information about MNI coordinates of each
  neural area depicted in the Schaefer atlas.

### Spatial Mapping and Coordinate Transformation

- In the following step, we mapped intensity values to spatial indices
  from the NIfTI image.
- Next, we transformed indices to MNI coordinates using the affine
  matrix.
- Finally, intensity values and corresponding MNI coordinates were
  saved in a CSV
  file.

### Mapping MNI Coordinates to AAL 3 Atlas

- Based on intensity values, MNI coordinates and intensities from the
  CSV file were
  merged with names of Schaefer cortical regions into a single file.
- In the last step, we used `label4MRI` function in mri.std.prepar.script.Rmd to
  extract AAL 3 labels based on MNI coordinates and we relabeled names of
  regions using AAL 3.
