# Supplementary material for: Predicting the degree of trait emotional empathy from cortical features using surface-based morphometry
Source: Sci Rep. 2026 Mar 25;16:14893. doi: 10.1038/s41598-026-44137-9 (PMC13168272; doi:10.1038/s41598-026-44137-9)

## Left insula

Supplementary Table 1: Model fit of multiple regression analysis using non-linear terms for prediction degree of empathy based on cortical thickness in left insula

| Model | coefficient | standard_error | t_value | p_value | BIC |
| --- | --- | --- | --- | --- | --- |
| Linear | -4.93 | 2.06 | -2.40 | 0.020 | 318.8151 |
| Quadratic | -19.23 | 8.08 | -2.38 | 0.021 | 317.2592 |
| Cubic | -15.04 | 35.13 | -0.43 | 0.670 | 321.1908 |
| Quartic | -118.77 | 171.62 | -0.69 | 0.492 | 324.7991 |
| Note. BIC = Bayesian information criterion | | | | | |

Supplementary Table 2: Model fit of non-linear mixed effect model predicting degree of empathy based on cortical thickness in left insula

| Model | coefficient | standard_error | t_value | p_value | BIC |
| --- | --- | --- | --- | --- | --- |
| Linear | -4.72 | 1.96 | -2.41 | 0.019 | 319.8002 |
| Quadratic | -16.89 | 8.71 | -1.94 | 0.057 | 319.9626 |
| Cubic | -22.39 | 33.07 | -0.68 | 0.501 | 323.5844 |
| Quartic | -134.13 | 162.21 | -0.83 | 0.412 | 326.9455 |
| Note. BIC = Bayesian information criterion | | | | | |

## Right insula

Supplementary Table 3: Results of multiple regression analysis using non-linear terms for prediction degree of empathy based on cortical thickness in right insula

| Model | coefficient | standard_error | t_value | p_value | BIC |
| --- | --- | --- | --- | --- | --- |
| Linear | -0.53 | 2.15 | -0.25 | 0.805 | 324.4294 |
| Quadratic | 5.51 | 9.50 | 0.58 | 0.564 | 328.2043 |
| Cubic | -13.22 | 42.13 | -0.31 | 0.755 | 332.2264 |
| Quartic | -92.92 | 209.04 | -0.44 | 0.658 | 336.1389 |
| Note. BIC = Bayesian information criterion | | | | | |

Supplementary Table 4: Model fit of non-linear mixed effect model predicting degree of empathy based on cortical thickness in right insula

| Model | coefficient | standard_error | t_value | p_value | BIC |
| --- | --- | --- | --- | --- | --- |
| Linear | -0.64 | 2.17 | -0.29 | 0.770 | 325.6081 |
| Quadratic | 10.08 | 8.93 | 1.13 | 0.264 | 328.3659 |
| Cubic | 5.83 | 39.92 | 0.15 | 0.884 | 332.4694 |
| Quartic | -24.28 | 196.93 | -0.12 | 0.902 | 336.5794 |
| Note. BIC = Bayesian information criterion | | | | | |

## Left ACC

Supplementary Table 5: Results of multiple linear regression predicting degree of empathy based on cortical thickness in left ACC

| Model | coefficient | standard_error | t_value | p_value | BIC |
| --- | --- | --- | --- | --- | --- |
| Linear | -4.51 | 2.07 | -2.18 | 0.033 | 319.7714 |
| Quadratic | 4.53 | 7.85 | 0.58 | 0.566 | 323.5498 |
| Cubic | -9.49 | 30.87 | -0.31 | 0.760 | 327.5760 |
| Quartic | -43.26 | 139.82 | -0.31 | 0.758 | 331.5991 |
| Note. BIC = Bayesian information criterion, ACC = Anterior Cingulate Cortex | | | | | |

Supplementary Table 6: Results of non-linear mixed effect model predicting degree of empathy based on cortical thickness in left ACC

| Model | coefficient | standard_error | t_value | p_value | BIC |
| --- | --- | --- | --- | --- | --- |
| Linear | -5.47 | 2.00 | -2.73 | 0.008 | 318.2123 |
| Quadratic | 1.58 | 7.23 | 0.22 | 0.828 | 322.2875 |
| Cubic | -14.33 | 28.15 | -0.51 | 0.613 | 326.1285 |
| Quartic | -140.93 | 129.70 | -1.09 | 0.282 | 328.9389 |
| Note. BIC = Bayesian information criterion, ACC = Anterior Cingulate Cortex | | | | | |

Supplementary Table 7: Results from LOO cross-validation

| Measure | RMSEP | R2_fitted | Q2 | Q2_perf |
| --- | --- | --- | --- | --- |
| thickness | 3.25 | 0.30 | -0.18 | -8.73 |
| gyrification | 3.48 | 0.60 | -0.35 | -9.20 |
| depth | 3.20 | 0.31 | -0.14 | -9.31 |
| RMSEP = Root Mean Square Error of Prediction, Q2 = Cross-Validated R-Squared, LOO = leave-one-out | | | | |

Supplementary Table 8: Correlation table depicting associations between empathy, cortical thickness of surface neural areas and age

| Variables | 1 | 2 | 3 | 4 | M(SD) |
| --- | --- | --- | --- | --- | --- |
| 1. TEQ | - |  |  |  | 21.90 (3.02) |
| 2. age | -.12 | - |  |  | 30.55 (12.02) |
| 3. Left_insula | -.26* | -.24 | - |  | 2.66 (0.18) |
| 4. Right_insula | .00 | -.31* | .28* | - | 2.89 (0.18) |
| 5. Left_ACC | -.28* | -.33** | .48*** | .34** | 2.89 (0.18) |
| Note. * p < 0.05; ** p < 0.01; *** p < 0.001; SD = standard deviation, M = mean, TEQ = Toronto Empathy Questionnaire, ACC = Anterior Cingulate Cortex, Spearman correlation coefficient was used. | | | | | |


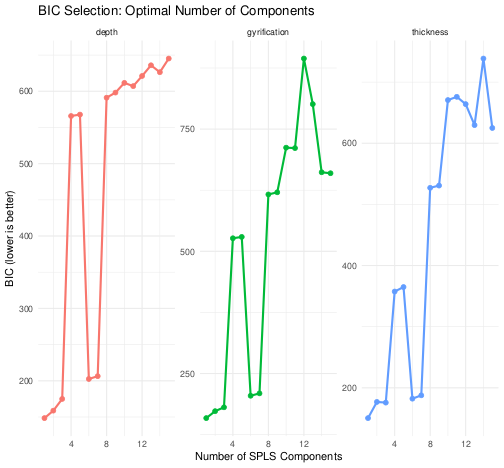


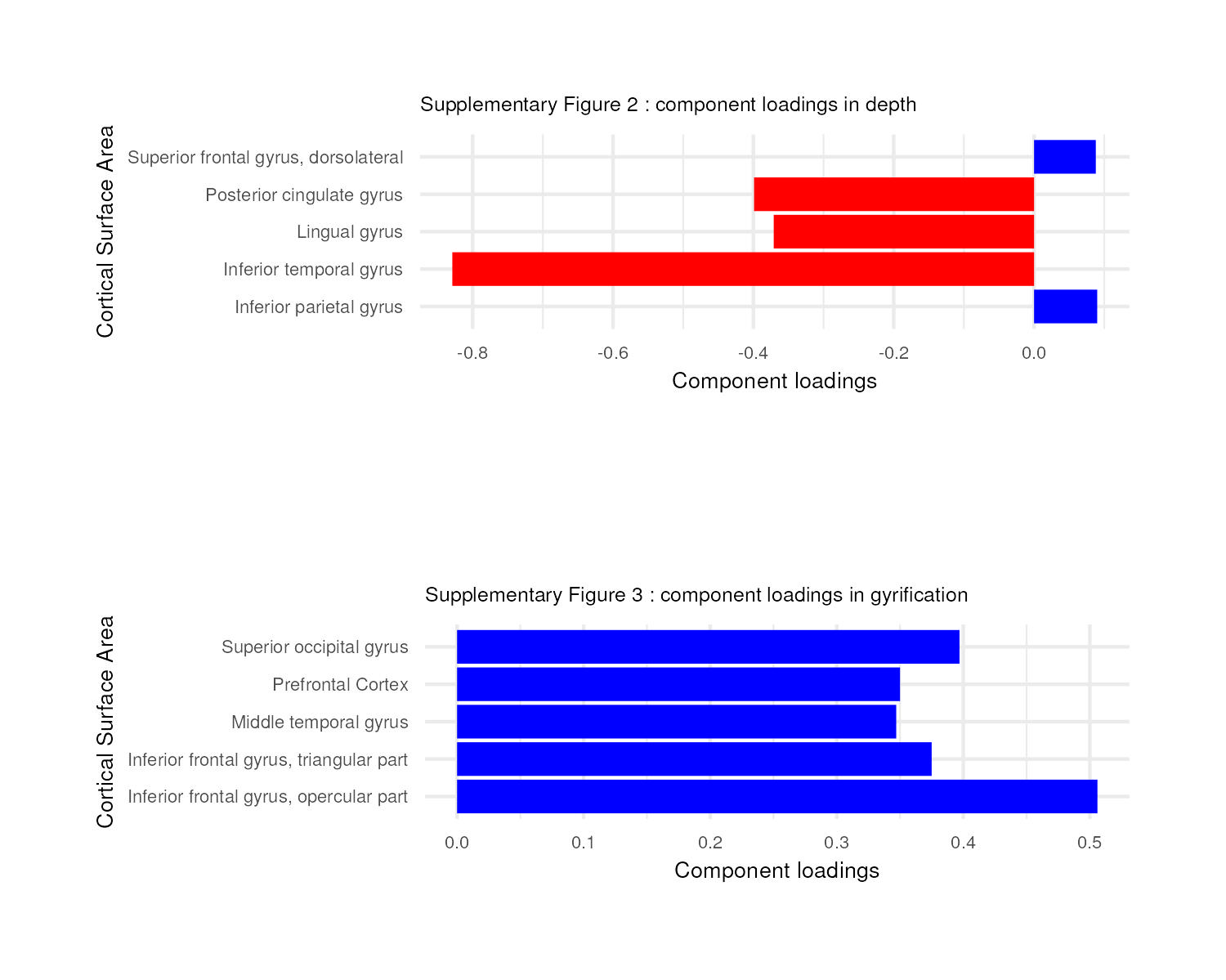


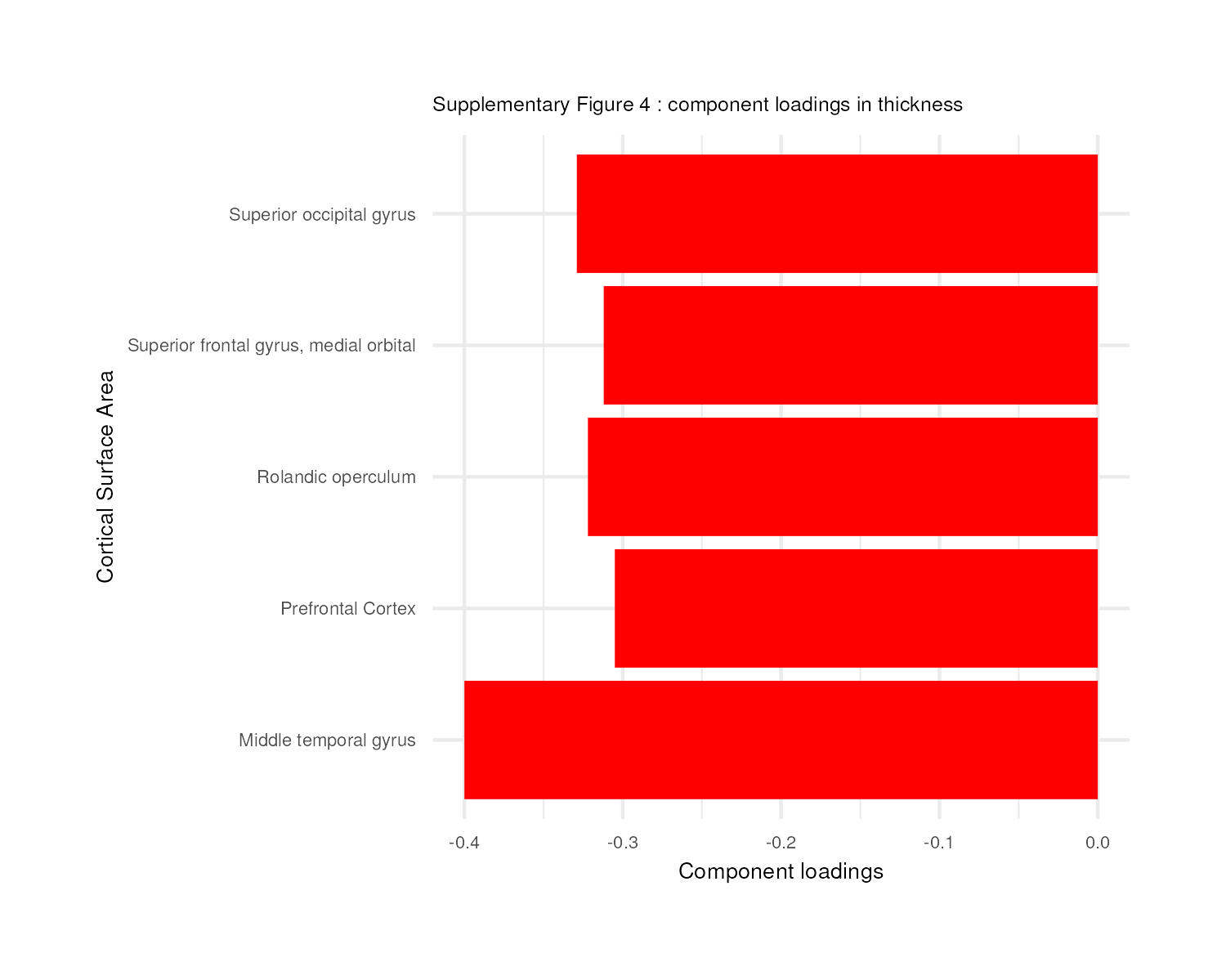

Supplement: Supplementary file 1 — Supplementary Material 1 [file 41598_2026_44137_MOESM1_ESM.zip › Supplementary_Material_2.docx]
